# Postoperative pulmonary rehabilitation compliance among patients with lung cancer: a cross-sectional survey

**DOI:** 10.3389/fonc.2025.1687014

**Published:** 2026-01-27

**Authors:** Xiangting Hu, Fengqiu Sun, Lingyan Jiang

**Affiliations:** 1 Department of Nursing, The Fourth Affiliated Hospital of Soochow University, Suzhou, Jiangsu, China; 2 Department of Thoracic and Cardiovascular Surgery, The Fourth Affiliated Hospital of Soochow University, Suzhou, Jiangsu, China

**Keywords:** pulmonary rehabilitation, compliance, lung cancer, nursing, care

## Abstract

**Background:**

Pulmonary rehabilitation plays a pivotal role in optimizing post-operative recovery outcomes for patients with lung cancer; however, suboptimal compliance continues to pose a significant barrier to its clinical effectiveness. This study aimed to assess the current status of postoperative pulmonary rehabilitation compliance among lung cancer patients and identify the key influencing factors.

**Method:**

A cross-sectional survey was conducted with lung cancer patients who underwent surgery, using a validated scale to assess pulmonary rehabilitation compliance. Correlation analysis and multivariate linear regression analysis were conducted to evaluate the influencing factors.

**Results:**

A total of 262 lung cancer patients were enrolled in this study (response rate 87.9%, as detailed in **Supplementary File 1**). The overall pulmonary rehabilitation compliance score was moderate, at 57.08 ± 9.84, with significant differences across three dimensions; notably, “active advice-seeking” had the lowest average item score (3.48 ± 1.04). Correlation analysis showed that compliance was strongly associated with age (*r* = -0.621, *p* = 0.024), educational level (*r* = -0.598, *p* = 0.017), marital status (*r* = 0.602, *p* = 0.040), place of residence (*r* = 0.647, *p* = 0.001), average monthly household income per capita (*r* = -0.591, *p* = 0.031), and lung cancer histological type (*r* = -0.574, *p* = 0.045). Multivariate linear regression analysis identified independent predictors of poorer compliance, including older age, lower educational level, unmarried/widowed/divorced status, rural residence, lower household income, and specific lung cancer classifications. The regression model demonstrated good fit (*R*²=0.591, *F* = 28.558, *p* < 0.001).

**Conclusion:**

Postoperative pulmonary rehabilitation adherence among lung cancer patients still has considerable room for improvement, highlighting the need for multi-dimensional, targeted strategies to enhance patient compliance.

## Introduction

Lung cancer, as one of the malignant tumors with the highest incidence and mortality worldwide, surgical resection remains the core treatment modality offering a curative opportunity for patients in the early and middle stages (1). However, postoperative issues such as impaired pulmonary function, decreased respiratory muscle strength, and reduced exercise endurance are prevalent. These not only diminish patients’ quality of life but also potentially increase the risk of postoperative complications, affecting long-term prognosis (2). Pulmonary rehabilitation, as a comprehensive multidisciplinary intervention, encompasses structured exercise training, respiratory function exercises, nutritional support, and psychological interventions (3). It has been proven to effectively improve pulmonary function, alleviate dyspnea symptoms, and enhance exercise capacity and quality of life in post-operative lung cancer patients, thus becoming an essential component of postoperative continuity of care (4, 5). Nevertheless, clinical practice indicates that patient compliance with pulmonary rehabilitation programs is generally low, with approximately 30%-50% of patients failing to adhere to the rehabilitation plan, resulting in the inability to achieve the expected therapeutic effects (6). Therefore, clarifying the current status of pulmonary rehabilitation compliance among post-operative lung cancer patients and its underlying influencing mechanisms is a prerequisite for optimizing rehabilitation interventions and improving clinical benefits.

In recent years, while the clinical value of pulmonary rehabilitation in postoperative lung cancer management has been widely acknowledged, its broader implementation is hindered by a pivotal bottleneck: inadequate patient compliance. Existing research (7, 8) indicates that postoperative pulmonary rehabilitation compliance among lung cancer patients is shaped by the interplay of multi-dimensional factors. At the individual level, patients may develop exercise-related fears due to postoperative physical discomfort (e.g., pain, fatigue) or disregard long-term adherence owing to insufficient awareness of rehabilitation’s clinical significance. At the social support level, the involvement of family caregivers and the quality of follow-up care provided by healthcare teams directly impact the continuity of patients’ rehabilitation practices. At the healthcare system level, factors such as the individualization of rehabilitation programs and the accessibility of community-based rehabilitation resources further constrain compliant behaviors. However, most current studies on postoperative lung cancer patients focus primarily on validating the efficacy of pulmonary rehabilitation, with insufficient systematic characterization of compliance status (9). Furthermore, analyses of influencing factors are often confined to single dimensions, lacking in-depth exploration of multi-factor interactions—resulting in a paucity of robust evidence to inform the development of targeted intervention strategies.

With the advent of the “whole-course management” era in cancer care, enhancing the quality of postoperative rehabilitation and extending patients’ survival have emerged as core objectives of comprehensive lung cancer treatment—where pulmonary rehabilitation compliance serves as the critical bridge linking medical interventions to tangible patient benefits (10). Currently, the standardized implementation of postoperative pulmonary rehabilitation for lung cancer in China remains in its nascent stage (11). Significant variations exist in rehabilitation program design and follow-up management models across different medical institutions, which further amplify individual and regional disparities in patient compliance (12). Meanwhile, existing nursing strategies are predominantly rooted in “one-size-fits-all guidance,” failing to fully integrate patients’ individual characteristics and practical needs, thereby limiting their intervention efficacy. Against this backdrop, the present study aims to systematically investigate the current status of postoperative pulmonary rehabilitation compliance among lung cancer patients, scientifically identify its key influencing factors, provide a robust evidence base for targeted clinical interventions, and promote the standardization and refinement of postoperative rehabilitation nursing for lung cancer.

## Methods

### Study design

This study adopts a cross-sectional survey design, with data collected via standardized questionnaires. It aims to objectively investigate the current status of pulmonary rehabilitation compliance among patients after lung cancer surgery, identify influencing factors, and propose targeted nursing strategies accordingly.

### Ethical considerations

This study has obtained approval from the ethics committee of The Fourth Affiliated Hospital of Soochow University (approval number: 220095). All patients signed written informed consent forms. During the research process, strict measures were taken to protect patient privacy: questionnaires were labeled only with serial numbers without recording personal identifiers such as names; data were stored on encrypted computers with access restricted exclusively to the research team; patients were entitled to withdraw from the study at any time without affecting their routine diagnosis and treatment services.

### Sample size estimation

Based on the sample size calculation formula for multivariate analysis (n = k × (10–15), where k is the number of independent variables) (13), combined with pre-surveys and literature reviews, this study included 14 independent variables, including demographic characteristics, disease-related indicators, rehabilitation cognition, and social support. Using the maximum coefficient of 15, the calculated sample size was n = 14 × 15 = 210 cases. Considering a 10% invalid or dropout rate, this study required at least 231 patients to meet the statistical requirements for multivariate regression analysis.

### Study participants

#### Sampling frame and methodology

Consecutive sampling was employed to recruit participants from patients who underwent lung cancer surgery (lobectomy, segmentectomy, or wedge resection) in the Department of Thoracic Surgery of our hospital between March 2023 and May 2025. A total of 298 patients were initially identified as eligible based on the predefined inclusion and exclusion criteria. Among these, 262 patients agreed to participate in the study (response rate = 87.9%), while 36 declined enrollment (12.1%). The primary reasons for refusal were time constraints (n=21) and reluctance to complete the study questionnaires (n=15). No systematic exclusions were applied beyond the predefined criteria, ensuring minimal selection bias.

#### Inclusion criteria

(1) Confirmed diagnosis of primary lung cancer via pathological examination and receipt of elective surgical treatment; (2) Postoperative survival time ≥ 1 month with stable clinical status (i.e., no severe postoperative complications such as persistent pleural effusion or infection); (3) Clear consciousness and ability to comprehend study procedures; (4) Voluntary participation in the study and provision of written informed consent.

#### Exclusion criteria

(1) Comorbidity with severe cardiovascular and cerebrovascular diseases, liver or kidney failure, or other medical conditions that could interfere with rehabilitation training; (2) Presence of cognitive impairment, mental illness, or communication disorders precluding effective completion of questionnaires; (3) Experience of severe adverse reactions (e.g., grade III or above myelosuppression) during postoperative adjuvant therapy (e.g., radiotherapy, chemotherapy).

### Study instruments

A comprehensive questionnaire was developed by combining a self-designed general information questionnaire with mature scales, consisting of two parts:

#### General information questionnaire

Included demographic characteristics (gender, age, educational level, marital status, place of residence, occupational status, average monthly household income per capita, medical expense payment method) and disease-related information (smoking history, surgical method, location of lesion resection, clinical stage, postoperative time, presence of comorbid chronic diseases (chronic obstructive pulmonary disease, COPD)).It was used to describe the baseline characteristics of the sample and serve as potential influencing factors for analysis.

#### Pulmonary rehabilitation compliance assessment scale

The scale used in this study was an adapted version of the previously reported Weng Exercise Compliance Scale (14), modified specifically to align with the postoperative pulmonary rehabilitation context of lung cancer patients. Item adaptation was conducted by a panel of three thoracic surgery specialists and two rehabilitation therapists, who revised 4 original items to enhance clinical relevance—for example, replacing general terms such as “general exercise” with context-specific phrasing (“pulmonary rehabilitation exercises”) and incorporating items related to respiratory function training (a core component of postoperative pulmonary rehabilitation for lung cancer). Since the original scale was developed in Chinese, a rigorous translation/back-translation process was performed to ensure semantic consistency: two bilingual researchers independently conducted forward translation (Chinese → English), followed by back-translation (English → Chinese) by an independent bilingual expert unfamiliar with the original scale. Discrepancies identified during translation were resolved through group discussion involving the research team and clinical experts.

Prior to formal data collection, a pilot study was conducted with 30 postoperative lung cancer patients (not included in the final sample) to evaluate the scale’s clarity, feasibility, and acceptability. Based on feedback from pilot participants—particularly those with low literacy levels—minor adjustments were made to simplify complex sentence structures and enhance readability.

The adapted scale retains 15 items across three dimensions: ① Physical exercise compliance (6 items, e.g., “Able to perform daily pulmonary rehabilitation exercises as instructed by medical staff” and “Complete respiratory preparation before each exercise”); ② Exercise monitoring compliance (4 items, e.g., “Monitor own respiratory rate or blood oxygen saturation during exercise” and “Record the duration and feelings of each rehabilitation training session”); ③ Active advice-seeking compliance (5 items, e.g., “Proactively consult medical staff when encountering rehabilitation difficulties” and “Communicate rehabilitation experience with other postoperative patients”). Responses were scored using a 5-point Likert scale (1 = completely unable to do, 2 = mostly unable to do, 3 = half able to do, 4 = mostly able to do, 5 = completely able to do), with a total score ranging from 15 to 75; higher scores indicate better pulmonary rehabilitation compliance (15). The psychometric properties of the adapted scale were rigorously evaluated: for reliability, pre-survey results demonstrated a Cronbach’s α coefficient of 0.91 for the total scale and 0.83–0.88 for the three dimensions, indicating excellent internal consistency; for content validity, five experts (3 thoracic surgeons, 1 rehabilitation therapist, and 1 methodologist) assessed each item using a 4-point Likert scale (1 = irrelevant, 2 = slightly relevant, 3 = moderately relevant, 4 = highly relevant), yielding an item-level content validity index (I-CVI) of 0.80–1.00 and a scale-level content validity index (S-CVI) of 0.92, confirming high content validity; and for construct validity, exploratory factor analysis (EFA) with principal component analysis and varimax rotation was conducted on the study sample, extracting three factors that collectively explained 68.3% of the total variance, with all item factor loadings ranging from 0.65 to 0.89 (exceeding the standard threshold of 0.60), thus verifying good construct validity of the adapted scale for assessing pulmonary rehabilitation compliance in postoperative lung cancer patients.

### Data collection

Three uniformly trained researchers (all thoracic surgery nurses with more than 5 years of clinical experience) were responsible for questionnaire distribution and data collection. Prior to formal data collection, the research team underwent standardized training, which included reviewing study protocols, practicing administration of the questionnaire, and verifying inter-rater consistency: ten pilot questionnaires were independently rated by the three researchers, with a Cohen’s kappa coefficient of 0.86 indicating excellent inter-rater reliability. To minimize interviewer bias—particularly for patients with limited reading and writing abilities—validated, standardized scripts were used for all interactions, including uniform wording for explaining the study purpose, reading questionnaire items, and recording responses; these scripts were pre-tested in the pilot study to ensure neutrality and avoid leading language. Additionally, researchers were blinded to patients’ clinical outcomes (e.g., postoperative complications, survival status) to prevent outcome-related bias in data collection.

The survey was conducted during patients’ 1-month postoperative follow-up visit, either in a quiet consulting room or a private area adjacent to the ward (to ensure confidentiality during bedside data collection). Before initiating the survey, researchers provided a detailed explanation of the study objectives, questionnaire completion procedures, and privacy protection measures to each patient, obtaining written informed consent prior to participation. For patients capable of independent completion, paper questionnaires were distributed with standardized instructions (e.g., “Please select the option that best reflects your actual situation over the past month following surgery”), and completed questionnaires were collected on-site. For patients with limited literacy or reading/writing difficulties, researchers read each item aloud verbatim using the standardized script and recorded responses based on the patients’ oral answers to ensure the authenticity of information. All completed questionnaires were checked for completeness immediately after collection, with researchers prompting patients to supplement or correct missing or ambiguous items to ensure data validity. To maintain confidentiality, patient responses were not discussed with ward staff or family members, and completed questionnaires were sealed in opaque envelopes immediately following review.

### Data analysis

Data entry was performed using EpiData 3.1 software with double-independent entry and logical verification for accuracy, and statistical analyses were conducted via SPSS 26.0. Measurement data (e.g., age, total pulmonary rehabilitation compliance score) were expressed as mean ± standard deviation and categorical data (e.g., gender, surgical method) as frequencies and percentages (n, %); inter-group comparisons of measurement data used independent sample t-tests or one-way ANOVA, and categorical data used χ² tests to screen variables with P < 0.10 for multivariate analysis. Pearson or Spearman correlation analysis explored associations between compliance scores and patient characteristics, and a multiple linear regression model (α-in = 0.05; α-out = 0.10) identified independent influencing factors—with the ordinal Likert-sum compliance score (range: 32–72; skewness = -0.12, kurtosis = 0.08, approximating normality) deemed appropriate for linear regression as it reflects a continuous latent construct (compliance level), consistent with prior validation in similar scales.

Pre-regression assumption verification included linearity (assessed via scatter plots), residual normality (Q-Q plots, Shapiro-Wilk test p=0.231), and homoscedasticity (residual plots, Breusch-Pagan test p=0.187). Multicollinearity was evaluated using Variance Inflation Factor (VIF: 1.12–1.35) and Tolerance (0.74–0.89), with all values meeting the criteria (VIF < 2, Tolerance > 0.5) indicating no severe multicollinearity. On-site questionnaire completeness checks ensured no missing data, and model validity was confirmed via residual and influence diagnostics (Cook’s distance, leverage values) with no outliers identified. The final regression model showed good fit (R² = 0.591, F = 28.558, p < 0.001), explaining 59.1% of the variance in rehabilitation compliance. A P-value < 0.05 was considered statistically significant.

## Results

As shown in **Supplementary File 1**, a total of 298 patients were initially identified as eligible based on the predefined inclusion and exclusion criteria. Among these, 262 patients agreed to participate in the study (response rate = 87.9%), while 36 declined enrollment (12.1%). The primary reasons for refusal were time constraints (n=21) and reluctance to complete the study questionnaires (n=15). Finally, a total of 262 eligible lung cancer patients were included in this study.

The baseline characteristics of included patients and compliance associations are detailed in **Table 1A** (sociodemographic) and **Table 1B** (clinical). Among participants, 32 (12.2%) had comorbid COPD, and 48 (18.3%) had initiated adjuvant therapy.

**Table 1A:** Sociodemographic characteristics of patients with lung cancer (n=262)​.

Characteristic​	Categories​	Cases (%)​	Pulmonary rehabilitation compliance score [mean (95% CI)]​	Statistic (t/F)​	Effect size​	p
Gender​	Male​	158 (60.30%)​	56.01 (53.98–58.04)​	5.396​	Cohen’s d=0.32​	0.105​
​	Female​	104 (39.70%)​	59.24 (57.12–61.36)​	​	​	​
Age (years)​	<60​	148 (56.49%)​	60.17 (58.23–62.11)​	6.453​	Cohen’s d=0.82​	*0.013​
​	≥60​	114 (43.51%)​	52.06 (50.09–54.03)​	​	​	​
Educational level​	Junior high school or below​	146 (55.73%)​	51.19 (49.25–53.13)​	2.004​	η²=0.18​	**0.009​
​	Senior high school​	77 (29.39%)​	58.62 (56.31–60.93)​	​	​	​
​	College or above​	39 (14.88%)​	62.31 (59.47–65.15)​	​	​	​
Marital Status​	Married​	215 (82.06%)​	59.10 (57.45–60.75)​	1.163​	η²=0.15​	*0.014​
​	Unmarried​	9 (3.44%)​	55.27 (50.83–59.71)​	​	​	​
​	Divorced​	23 (8.78%)​	53.09 (50.01–56.17)​	​	​	​
​	Widowed​	15 (5.73%)​	50.12 (46.58–53.66)​	​	​	​
Place of residence​	Rural area​	90 (34.35%)​	53.94 (51.72–56.16)​	6.056​	Cohen’s d=0.68​	*0.025​
​	Urban area​	172 (65.65%)​	60.62 (59.03–62.21)​	​	​	​
Occupation Status​	Employed​	154 (58.78%)​	57.05 (55.18–58.92)​	5.744​	Cohen’s d=0.25​	0.108​
​	Unemployed​	108 (41.22%)​	59.58 (57.46–61.70)​	​	​	​
Average monthly household income per capita (CNY)​	<5000​	134 (51.15%)​	51.30 (49.37–53.23)​	6.828​	Cohen’s d=1.09​	**0.004​
​	≥5000​	128 (48.85%)​	62.46 (60.51–64.41)​	​	​	​
Medical expense payment method​	Public medical insurance​	220 (83.97%)​	57.89 (56.36–59.42)​	1.055​	η²=0.03​	0.087​
​	Commercial insurance​	18 (6.87%)​	60.33 (56.79–63.87)​	​	​	​
​	Self-payment​	24 (9.16%)​	55.21 (51.98–58.44)​	​	​	​

CI, Confidence Interval; CNY,Chinese Yuan; Cohen’s d for binary variables (0.2=small, 0.5=moderate, 0.8=large effect); η² for categorical variables (0.01=small, 0.06=moderate, 0.14=large effect); *p<0.05, **p<0.01.​​

**Table 1B:** Clinical characteristics of patients with lung cancer (n=262)​.

Characteristic​	Categories​	Cases (%)​	Pulmonary rehabilitation compliance score [mean (95% CI)]​	Statistic (t/F)​	Effect size​	p
History of smoking​	Yes​	136 (51.91%)​	55.17 (53.21–57.13)​	5.923​	Cohen’s d=0.38​	0.190​
​	No​	126 (48.09%)​	59.01 (56.98–61.04)​	​	​	​
Time since lung cancer diagnosis​	<1 month​	155 (59.16%)​	58.36 (56.54–60.18)​	2.003​	η²=0.02​	0.115​
​	1~6 months​	87 (33.21%)​	57.03 (54.67–59.39)​	​	​	​
​	>6 months​	20 (7.63%)​	55.12 (51.53–58.71)​	​	​	​
Histological type​	Squamous cell carcinoma​	253 (96.56%)​	57.19 (55.82–58.56)​	6.232​	Cohen’s d=0.15​	0.103​
​	Adenocarcinoma​	9 (3.44%)​	55.75 (50.92–60.58)​	​	​	​
Clinical stage of lung cancer​	Stage I​	208 (79.39%)​	58.82 (57.35–60.29)​	1.284​	η²=0.04​	*0.040​
​	Stage II​	52 (19.85%)​	56.34 (53.68–59.00)​	​	​	​
​	Stage III​	2 (0.76%)​	52.05 (48.21–55.89)​	​	​	​
Location of lesion resection​	Left upper lobe​	94 (35.88%)​	57.58 (55.31–59.85)​	1.881​	η²=0.03​	0.067​
​	Left lower lobe​	19 (7.25%)​	54.43 (50.61–58.25)​	​	​	​
​	Right upper lobe​	66 (25.19%)​	57.82 (55.27–60.37)​	​	​	​
​	Right middle lobe​	15 (5.73%)​	55.09 (51.24–58.94)​	​	​	​
​	Right lower lobe​	68 (25.95%)​	56.97 (54.56–59.38)​	​	​	​
Complicated with chronic diseases (hypertension/diabetes/hyperlipidemia)​	Yes​	88 (33.59%)​	58.50 (56.18–60.82)​	6.382​	Cohen’s d=0.16​	0.109​
​	No​	174 (66.41%)​	56.86 (55.23–58.49)​	​	​	​
Complicated with COPD​	Yes​	32 (12.21%)​	56.73 (53.51–59.95)​	0.927​	Cohen’s d=0.08​	0.346​
​	No​	230 (87.79%)​	57.12 (55.78–58.46)​	​	​	​
Adjuvant therapy status​	Yes​	48 (18.32%)​	54.92 (52.01–57.83)​	4.159​	Cohen’s d=0.22​	0.063​
​	No​	214 (81.68%)​	57.65 (56.27–59.03)​	​	​	​

​COPD,Chronic Obstructive Pulmonary Disease; Cohen’s d for binary variables (0.2=small, 0.5=moderate, 0.8=large effect); η² for categorical variables (0.01=small, 0.06=moderate, 0.14=large effect); *p<0.05.

Sociodemographic and clinical factors showed significant associations with compliance: younger age, higher education, married status, urban residence, higher income, and earlier clinical stage were all linked to better compliance (all p < 0.05; **Tables 1A**, **1B**). No significant differences were observed for gender, occupation, medical payment method, smoking history, lesion resection location, or comorbid chronic diseases (all p> 0.05).

**Table 2** showed overall pulmonary rehabilitation compliance was moderate (mean item score: 3.85 ± 1.01, 95% CI: 3.69–4.01), with “active advice-seeking” as the lowest-scoring dimension (3.48 ± 1.04). The scale demonstrated good internal consistency (Cronbach’s α = 0.91 for total scale, 0.83–0.88 for dimensions). Subgroup analyses (**Supplementary Table S1**) confirmed compliance varied by age, stage, and residence, aligning with univariate trends.

**Table 2:** Scores and psychometric properties of pulmonary rehabilitation compliance dimensions (n=262)​.

Dimension​	Number of items​	Total score [mean ± SD (min–max)]​	Average item score [mean ± SD (95% CI)]​	Compliance level¹​	Cronbach’s α​
Physical exercise compliance​	6​	22.45 ± 4.20 (12–30)​	3.78 ± 0.98 (3.60–3.96)​	Moderate​	0.86​
Exercise monitoring compliance​	4​	15.66 ± 3.07 (8–20)​	3.95 ± 0.95 (3.79–4.11)​	Moderate​	0.83​
Active seeking of advice compliance​	5​	17.18 ± 3.79 (5–25)​	3.48 ± 1.04 (3.30–3.66)​	Moderate​	0.88​
Total score​	15​	57.08 ± 9.84 (32–72)​	3.85 ± 1.01 (3.69–4.01)​	Moderate​	0.91​

​¹Compliance level definition: Low=≤3.0 points/item; Moderate=3.1–4.0 points/item; High=≥4.1 points/item; SD, Standard Deviation; CI, Confidence Interval; Cronbach’s α>0.8 indicates good internal consistency.​

Correlation analysis (**Table 3**) revealed strong associations between compliance and age, education, marital status, residence, income, and lung cancer type (|r/ρ > 0.5, all p < 0.05), with younger age, higher education, and urban residence predicting better compliance. No significant correlations were found with gender, occupation, or COPD (all p> 0.05).

**Table 3:** Correlation between pulmonary rehabilitation compliance score and patient characteristics (n=262)​.

Category​	Characteristic​	Correlation coefficient (test type)²​	95% CI​	Correlation strength³​	p
Sociodemographic​	Gender​	0.161 (Pearson’s r)​	(-0.012, 0.326)​	Weak​	0.093​
​	Age​	-0.621 (Pearson’s r)​	(-0.712, -0.508)​	Strong​	*0.024​
​	Educational level​	-0.598 (Spearman’s ρ)​	(-0.694, -0.481)​	Strong​	*0.017​
​	Marital Status​	0.602 (Spearman’s ρ)​	(0.485, 0.698)​	Strong​	*0.040​
​	Place of residence​	0.647 (Spearman’s ρ)​	(0.542, 0.733)​	Strong​	**0.001​
​	Occupation Status​	0.184 (Spearman’s ρ)​	(-0.003, 0.359)​	Weak​	0.096​
​	Average monthly household income per capita​	-0.591 (Pearson’s r)​	(-0.688, -0.473)​	Strong​	*0.031​
​	Medical expense payment method​	0.150 (Spearman’s ρ)​	(-0.025, 0.315)​	Weak​	0.116​
Clinical​	History of smoking​	0.108 (Spearman’s ρ)​	(-0.068, 0.276)​	Weak​	0.094​
​	Time since lung cancer diagnosis​	0.115 (Spearman’s ρ)​	(-0.061, 0.282)​	Weak​	0.103​
​	Histological type​	-0.574 (Spearman’s ρ)​	(-0.673, -0.452)​	Strong​	*0.045​
​	Clinical stage of lung cancer​	-0.489 (Spearman’s ρ)​	(-0.592, -0.365)​	Moderate​	*0.038​
​	Location of lesion resection​	0.119 (Spearman’s ρ)​	(-0.057, 0.286)​	Weak​	0.101​
​	Complicated with chronic diseases​	0.205 (Spearman’s ρ)​	(0.032, 0.368)​	Weak​	0.079​
​	Complicated with COPD​	0.095 (Pearson’s r)​	(-0.081, 0.263)​	Weak​	0.152​

​COPD,Chronic Obstructive Pulmonary Disease; ²Pearson’s r for normally distributed continuous variables; Spearman’s ρ for ordinal/categorical variables. ³Correlation strength(r/ρ): Weak= =0.1–0.3; Moderate=0.3–0.7; Strong=0.7–1.0. CI, Confidence Interval; COPD, Chronic Obstructive Pulmonary Disease; *p<0.05, **p<0.01.

Multivariate linear regression (**Table 4**; **Supplementary Figure 2**) identified independent predictors of compliance, with the model explaining 59.1% of variance (R² = 0.591, F = 28.558, p < 0.001). Older age, lower education, unmarried/widowed/divorced status, rural residence, lower income, and specific histological types were independently associated with poorer compliance (all p < 0.05). Multicollinearity diagnostics (VIF: 1.12–1.28) confirmed no severe model bias.

**Table 4:** Multivariate linear regression analysis of factors influencing pulmonary rehabilitation compliance score among patients with lung cancer (n=262).​.

Predictor​	Unstandardized β​	SE​	Standardized β​	95% confidence interval​	t​	p ​	VIF​	Tolerance​
Constant​	-150.06​	5.319​	-​	(-160.42, -139.70)​	-28.306​	0.034​	-​	-​
Age (years)​	-3.051​	1.406​	-0.092​	(-5.803, -0.299)​	-1.847​	*0.012​	1.12​	0.89​
Educational level​	-2.908​	1.161​	-0.106​	(-5.187, -0.629)​	-2.772​	*0.016​	1.35​	0.74​
Marital Status​	2.664​	1.009​	0.124​	(0.685, 4.643)​	2.150​	*0.037​	1.28​	0.78​
Place of residence​	3.676​	1.582​	0.130​	(0.571, 6.781)​	2.309​	**0.008​	1.19​	0.84​
Average monthly household income per capita (CNY)​	-3.035​	1.204​	-0.127​	(-5.401, -0.669)​	-2.115​	*0.010​	1.25​	0.80​
Classification of lung cancer​	-2.426​	1.179​	-0.112​	(-4.743, -0.109)​	-2.066​	*0.042​	1.18​	0.85​

Reference categories for categorical variables: Educational level (college or above vs. senior high school/junior high school or below); Marital status (married vs. unmarried/divorced/widowed); Place of residence (urban vs. rural); Average monthly household income per capita (≥5000 CNY vs. <5000 CNY); Classification of lung cancer (squamous cell carcinoma vs. adenocarcinoma); Significance markers: *p < 0.05, **p < 0.01. **Supplementary Figure 2** presents a forest plot of standardized β coefficients and 95% CIs for all influencing factors.

## Discussion

In the present study, postoperative pulmonary rehabilitation compliance among lung cancer patients was operationalized into three distinct dimensions: “exercise execution,” “symptom monitoring,” and “active advice-seeking.” The results revealed that overall compliance was moderate, with the “active advice-seeking” dimension yielding the lowest scores. This dimension not only reflects patients’ willingness to seek and utilize professional guidance but also predicts their capacity to access timely medical support when encountering unexpected symptoms or rehabilitation challenges. The low score in this domain indicates that while postoperative education effectively conveys the importance of “exercising” and “monitoring,” it fails to fully cultivate patients’ metacognitive strategies for determining “when and how to seek help.” In other words, the current rehabilitation model achieves knowledge transfer but falls short of fostering a patient-centered, continuous dialogue between patients and healthcare providers—highlighting a critical opportunity for optimizing subsequent intervention strategies (16).

The results of this study revealed that elderly patients, those with lower educational levels, the unmarried or widowed, rural residents, low-income individuals, and patients with higher-stage lung cancer exhibited significantly decreased postoperative pulmonary rehabilitation compliance. These factors do not exist in isolation but amplify step by step along the causal chain of “socioeconomic vulnerability → limited health cognition → weak support systems → difficulty in behavioral execution.” The decline in physiological reserve and comorbidities associated with advanced age reduce patients’ tolerance expectations for exercise prescriptions; low educational levels and limited access to information make it difficult for patients to translate abstract rehabilitation benefits into specific actions (17); the absence of marriage or family support weakens the mechanisms of reminder, companionship, and emotional reinforcement; rural and low-income backgrounds further exacerbate structural barriers such as transportation, equipment, and follow-up costs; patients with higher-stage diseases, due to severe symptom burden and poor prognosis perception, are prone to forming a fatalistic view of “rehabilitation ineffectiveness” and thus take the initiative to withdraw from the rehabilitation cycle (18, 19). It can be seen that compliance is not merely an individual choice but a multidimensional imbalance nested in the social-ecological system, suggesting that intervention design needs to go beyond the traditional paradigm of “education and supervision.” (20).

Notably, the interaction between socioeconomic vulnerability and advanced disease stages may exacerbate health inequities. Previous studies (21, 22) have confirmed that low-income and rural patients are disproportionately likely to present with stage III–IV lung cancer due to delayed initial diagnosis; in turn, advanced disease further impairs their economic and psychological capacity to engage in rehabilitation, creating a vicious cycle of “poverty → advanced stage → low compliance → poor prognosis.” Without targeted policy interventions, this cycle will continue to widen survival disparities between urban and rural populations, as well as across socioeconomic groups. Thus, pulmonary rehabilitation compliance should not be viewed merely as a marker of medical quality but rather integrated into “health equity” frameworks for systematic governance.

Traditional compliance management, centered on information provision and regular reminders, has proven ineffective for these vulnerable groups. Information alone does not translate into sustained behavioral change—especially when patients face multifaceted barriers such as transportation challenges, financial burdens, and emotional isolation (23–25). This necessitates expanding the nursing role beyond “knowledge disseminator” to encompass “resource linker” and “system coordinator” (26). For instance, a pre-discharge vulnerability screening could identify a “high-risk cohort” (including elderly patients, those living alone, rural residents, and those with advanced disease); interdisciplinary case management—integrating community nurses, rehabilitation therapists, social workers, and charitable resources—could then be initiated to develop tailored rehabilitation programs that are affordable, accessible, and sustainable for these patients (27–29).

Based on the study findings, we propose multi-level clinical nursing interventions tailored to address the identified barriers to pulmonary rehabilitation compliance: first, develop low-literacy-accessible educational packages integrating graphics and videos, paired with the teach-back method to verify patient understanding of rehabilitation protocols (30, 31); second, introduce wearable monitoring devices to deliver real-time feedback on exercise intensity, alleviating elderly patients’ fears of overexertion (32, 33); third, establish “satellite rehabilitation centers” in collaboration with primary health institutions, adopting a hybrid model of remote guidance and centralized training to bridge urban-rural disparities in rehabilitation access (34, 35); fourth, implement transportation subsidies and equipment sharing libraries to mitigate the economic burden on low-income patients; and fifth, construct a tripartite “patient-family-volunteer” support network, assigning “rehabilitation partners” to patients living alone or lacking family support to enhance adherence continuity (36–38).

This study has several limitations to consider when interpreting the results. First, the cross-sectional design only identifies correlations—not causal relationships—between pulmonary rehabilitation compliance and influencing factors, with potential confounding by unmeasured variables (e.g., health beliefs, information acquisition ability). Secondly, the sample was recruited from a single tertiary hospital, which may introduce selection bias. The representativeness of vulnerable subgroups—including rural patients, low-income individuals, and those with comorbid conditions such as chronic obstructive pulmonary disease (COPD)—is limited. Notably, the proportion of COPD patients in our sample was 12.2% (n=32), which is lower than the reported prevalence of 30%-40% in the broader cancer population (39, 40). This underrepresentation may have reduced statistical power to detect potential effects of COPD on compliance, contributing to the non-significant finding observed in the regression analysis. As such, the results cannot be directly generalized to patients treated in primary medical institutions, community hospitals, or other geographic regions. Third, compliance was assessed via self-reported data (first postoperative month) without objective verification (e.g., wearable devices), risking recall or social desirability bias; notably, 18.3% (n=48) of participants had initiated adjuvant therapy (radiotherapy/chemotherapy) at survey, and while subgroup size minimized overall impact, therapy-related side effects (e.g., fatigue) may have marginally reduced adherence in these patients. Fourth, no analyses of factor interactions (e.g., rural residence + low income) or in-depth mechanistic investigations were conducted, limiting insights into compliance behavior. Future multi-center cohort studies should enhance representativeness, integrate objective monitoring tools, incorporate mediation/moderation analyses to clarify causal pathways, and explore adjuvant therapy impacts across longer follow-up (e.g., weeks 2–12, when adherence fluctuates) to strengthen findings’ relevance and generalizability.

## Conclusion

In conclusion, this study demonstrates that postoperative pulmonary rehabilitation compliance among lung cancer patients remains moderate overall, with the “active advice-seeking” dimension yielding the lowest scores—underscoring a critical gap in current rehabilitation education: the failure to adequately cultivate patients’ awareness of and strategies for seeking timely help when facing rehabilitation challenges. Notably, key populations with poor adherence include older adults, individuals with lower educational attainment, the unmarried/widowed, rural residents, low-income groups, and patients with advanced-stage lung cancer. These factors operate through a cascading pathway: “socioeconomic vulnerability → limited health literacy → weakened support systems → barriers to behavioral implementation.” Furthermore, the interplay between socioeconomic status and disease stage may exacerbate health disparities, widening the gap in recovery outcomes across populations.

The findings highlight that improving postoperative pulmonary rehabilitation adherence requires moving beyond the traditional “one-size-fits-all guidance” model toward multi-dimensional, personalized interventions. In clinical practice, priority should be placed on stratified management of high-risk groups: visual educational tools tailored to low literacy levels (e.g., graphic-video combinations) can enhance health understanding; wearable devices providing real-time exercise intensity feedback can alleviate older patients’ fears of overexertion. Simultaneously, constructing a collaborative “medical-community-family” support network is essential—including establishing satellite rehabilitation hubs to bridge urban-rural resource inequities, offering transportation and equipment subsidies to reduce financial burdens, and pairing isolated patients with “rehabilitation buddies” to address social support deficits. Such comprehensive measures have the potential to effectively improve adherence, shifting pulmonary rehabilitation from mere “knowledge transfer” to sustained behavioral change, ultimately enhancing overall postoperative recovery outcomes and advancing health equity.

## Data Availability

The original contributions presented in the study are included in the article/**Supplementary Material**. Further inquiries can be directed to the corresponding authors.

## Supplementary material

Supplementary Figure 1 Patient enrollment flow diagram.

Supplementary Figure 2 Forest plot of standardized β coefficients for multivariate linear regression.
