# Supplementary figures and images for: Postoperative pulmonary rehabilitation compliance among patients with lung cancer: a cross-sectional survey
Source: Front Oncol. 2026 Jan 27;15:1687014. doi: 10.3389/fonc.2025.1687014 (PMC12888214; doi:10.3389/fonc.2025.1687014)

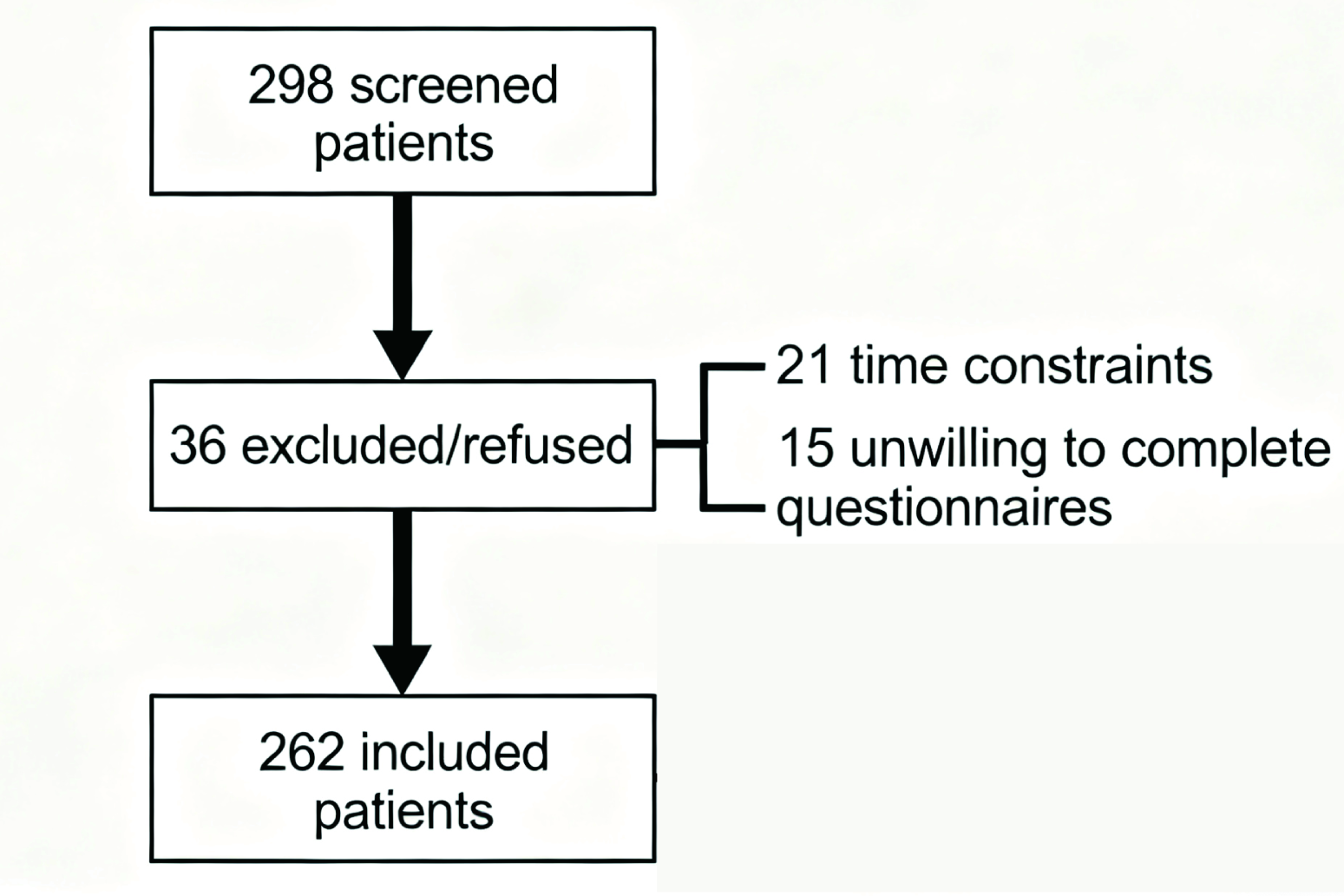

Supplement: Supplementary Figure 1 — Patient enrollment flow diagram. [file Image1.tif]

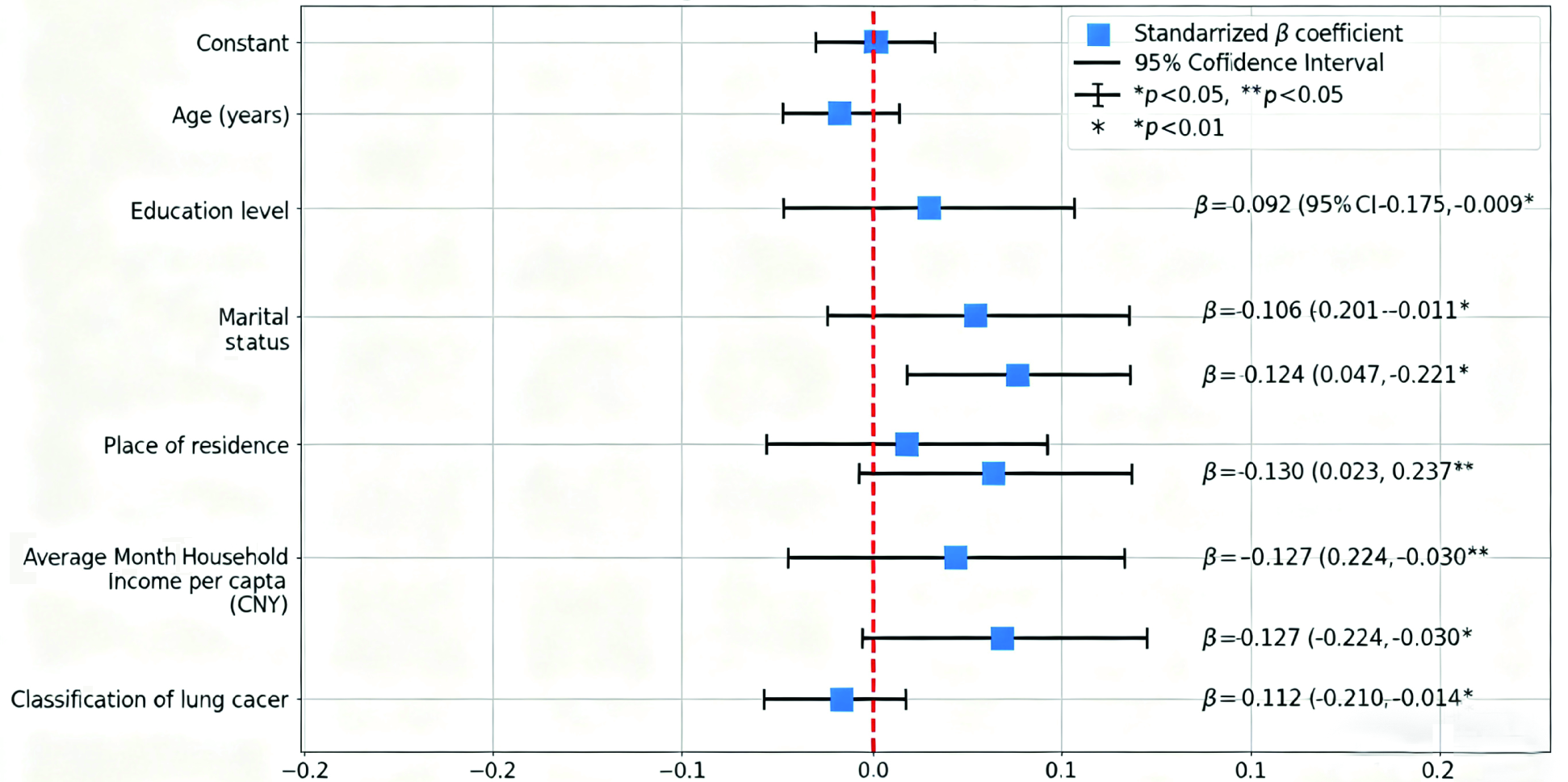

Supplement: Supplementary Figure 2 — Forest plot of standardized β coefficients for multivariate linear regression. [file Image2.tif]
