# Supplementary material for: Postoperative pulmonary rehabilitation compliance among patients with lung cancer: a cross-sectional survey
Source: Front Oncol. 2026 Jan 27;15:1687014. doi: 10.3389/fonc.2025.1687014 (PMC12888214; doi:10.3389/fonc.2025.1687014)
Supplement: Supplementary file 3 [file Table1.docx]

Supplementary Table 1 Pulmonary rehabilitation compliance dimension scores stratified by key variables (n=262)

| Key Variable | Subgroup | Cases (%) | Physical Exercise Compliance [Mean ± SD (95% CI)] | Exercise Monitoring Compliance [Mean ± SD (95% CI)] | Active Seeking of Advice Compliance [Mean ± SD (95% CI)] | Total Compliance [Mean ± SD (95% CI)] | Group Comparison (t/F) | p-value | Effect Size |
| --- | --- | --- | --- | --- | --- | --- | --- | --- | --- |
| Age (years) | <60 | 148 (56.49%) | 24.03±4.15 (23.21–24.85) | 16.52±2.98 (15.95–17.09) | 18.62±3.67 (17.94–19.30) | 60.17±10.44 (58.23–62.11) | 12.86 (t) | *0.013 | Cohen’s d=0.82 |
|  | ≥60 | 114 (43.51%) | 20.15±3.89 (19.32–20.98) | 14.38±3.01 (13.75–15.01) | 15.12±3.58 (14.37–15.87) | 52.06±9.57 (50.09–54.03) |  |  |  |
| Clinical Stage | Stage I | 208 (79.39%) | 23.12±4.08 (22.45–23.79) | 16.05±2.95 (15.58–16.52) | 17.65±3.69 (17.08–18.22) | 58.82±10.77 (57.35–60.29) | 3.52 (F) | *0.040 | η²=0.04 |
|  | Stage II | 52 (19.85%) | 21.56±4.21 (20.38–22.74) | 15.03±3.12 (14.18–15.88) | 16.21±3.81 (15.17–17.25) | 56.34±9.47 (53.68–59.00) |  |  |  |
|  | Stage III | 2 (0.76%) | 18.50±4.95 (13.55–23.45) | 13.00±4.24 (8.76–17.24) | 13.25±4.50 (8.75–17.75) | 52.05±9.06 (48.21–55.89) |  |  |  |
| Place of Residence | Urban area | 172 (65.65%) | 23.58±4.02 (22.91–24.25) | 16.31±2.92 (15.82–16.80) | 18.23±3.61 (17.64–18.82) | 60.62±10.24 (59.03–62.21) | 10.63 (t) | *0.025 | Cohen’s d=0.68 |
|  | Rural area | 90 (34.35%) | 20.52±4.18 (19.58–21.46) | 14.56±3.15 (13.83–15.29) | 15.68±3.72 (14.87–16.49) | 53.94±9.96 (51.72–56.16) |  |  |  |
| Average Monthly Household Income (CNY) | <5000 | 134 (51.15%) | 19.87±4.25 (18.98–20.76) | 14.22±3.21 (13.54–14.90) | 15.03±3.85 (14.25–15.81) | 51.30±10.46 (49.37–53.23) | 18.95 (t) | **0.004 | Cohen’s d=1.09 |
|  | ≥5000 | 128 (48.85%) | 25.21±3.98 (24.45–25.97) | 17.18±2.76 (16.65–17.71) | 19.45±3.42 (18.82–20.08) | 62.46±9.78 (60.51–64.41) |  |  |  |
| Marital Status | Married | 215 (82.06%) | 23.05±4.06 (22.41–23.69) | 15.98±2.97 (15.53–16.43) | 17.56±3.65 (17.02–18.10) | 59.10±10.95 (57.45–60.75) | 6.89 (F) | *0.014 | η²=0.15 |
|  | Unmarried | 9 (3.44%) | 21.33±4.32 (17.98–24.68) | 15.11±3.05 (12.86–17.36) | 16.00±3.92 (13.08–18.92) | 55.27±9.71 (50.83–59.71) |  |  |  |
|  | Divorced | 23 (8.78%) | 20.43±4.19 (18.65–22.21) | 14.61±3.20 (13.27–15.95) | 14.87±3.79 (13.31–16.43) | 53.09±9.88 (50.01–56.17) |  |  |  |
|  | Widowed | 15 (5.73%) | 19.27±3.98 (17.21–21.33) | 13.87±3.12 (12.18–15.56) | 14.00±3.64 (12.16–15.84) | 50.12±10.35 (46.58–53.66) |  |  |  |

Notes:

1. SD=Standard Deviation; CI=Confidence Interval; CNY=Chinese Yuan.
2. Compliance dimension definitions: Physical Exercise Compliance (6 items, total score range 6–30); Exercise Monitoring Compliance (4 items, total score range 4–20); Active Seeking of Advice Compliance (5 items, total score range 5–25); Total Compliance (15 items, total score range 15–75).
3. Compliance level criteria: Low=≤3.0 points/item; Moderate=3.1–4.0 points/item; High=≥4.1 points/item.
4. Statistical tests: Independent samples t-test (t) for binary variables; one-way ANOVA (F) for categorical variables with ≥3 subgroups.
5. Effect size interpretation: Cohen’s d (binary variables): 0.2=small, 0.5=moderate, 0.8=large effect; η² (categorical variables): 0.01=small, 0.06=moderate, 0.14=large effect.
6. Significance markers: *p<0.05, **p<0.01.
