# Supplementary material for: Postoperative pulmonary rehabilitation compliance among patients with lung cancer: a cross-sectional survey
Source: Front Oncol. 2026 Jan 27;15:1687014. doi: 10.3389/fonc.2025.1687014 (PMC12888214; doi:10.3389/fonc.2025.1687014)
Supplement: Supplementary file 4 [file DataSheet1.pdf]

Investigation on the Current Situation of Pulmonary Rehabilitation Compliance among Lung Cancer Patients after Surgery

Dear patient,

Hello! Our research team is engaged in the study of rehabilitation after lung cancer surgery. Currently, we are conducting a survey on the compliance of pulmonary rehabilitation among lung cancer patients after surgery. The purpose of this research is to gain a deeper understanding of your actual situation and needs during the postoperative pulmonary rehabilitation process, so as to provide you and patients with similar conditions with more scientific and practical nursing services and rehabilitation guidance.

This survey adopts a questionnaire format. The content is designed around the behaviors and feelings related to postoperative pulmonary rehabilitation. You just need to answer based on your actual situation after careful consideration. The whole process will take about 15 minutes.

Please rest assured that all the personal information and questionnaire content you provide will be strictly confidential. In the public report of the research results, we will conduct a comprehensive analysis of all the information, and we will never disclose any content that may identify your personal identity. We will do our best to protect your privacy.

Your participation is of great significance to this research. Your genuine feelings and feedback will provide valuable evidence for our research. At the same time, whether to participate in this survey is completely up to your own will. You have the right to withdraw at any time without being affected in any way.

If you are willing to participate in this survey, please sign below to confirm. Thank you for your support and help in your busy schedule. We wish you a speedy recovery!

I. Basic Demographic Information

1. Gender

- ◻ Male
- ◻ Female

2. Age (in years)

- ______________________________

3. Educational Attainment

- ◻ Junior high school or below
- ◻ High school
- ◻ College or above

4. Marital Status

- ◻ Married
- ◻ Single
- ◻ Divorced
- ◻ Widowed

5. Place of Residence

- ◻ City
- ◻ Rural area

6. Occupation Status
    - □ Farmer
    - □ Worker
    - □ Enterprise or institution staff
    - □ Unemployed
    - □ Retired
    - □ Others
7. Average Monthly Household Income per Capita
    - □ Less than 5,000 yuan
    - □ 5,000 yuan or more
8. Medical Expense Payment Method
    - □ Public medical insurance
    - □ Commercial medical insurance
    - □ Self - payment
    - □ Others
9. Smoking History

- ☐ Never smoked
- ☐ Quit smoking
- ☐ Smoking: ___________ cigarettes per day

10. Disease Diagnosis Time
    - ☐ Less than 1 month
    - ☐ 1 - 6 months
    - ☐ More than 6 months
11. Tissue Type
    - ☐ Squamous cell carcinoma
    - ☐ Adenocarcinoma
    - ☐ Others
12. Clinical Stage
    - ☐ Stage I
    - ☐ Stage II
    - ☐ Stage III
    - ☐ Stage IV

- ◻ Others

13. Location of Lesion Resection
    - ◻ Left upper lobe
    - ◻ Left lower lobe
    - ◻ Right upper lobe
    - ◻ Right middle lobe
    - ◻ Right lower lobe
14. Surgical Method
    - ◻ Lobectomy
    - ◻ Segmentectomy
    - ◻ Wedge resection
    - ◻ Others
15. Whether Complicated with Chronic Diseases (Hypertension/Diabetes/Hyperlipidemia)
    - ◻ No
    - ◻ Yes: ___________

II. Pulmonary Rehabilitation Exercise Compliance Scale

Dimension	Serial Number	Item Content	Can't Do at All (1 point)	Mostly Can't Do (2 points)	Can Do Half (3 points)	Mostly Can Do (4 points)	Can Do Completely (5 points)
Physical Exercise Compliance	1	Can remember the specific methods of exercise training.	□	□	□	□	□
	2	Can exercise every day according to the guidance of medical staff.	□	□	□	□	□
	3	Can exercise 3 - 4 times a day as required.	□	□	□	□	□
	4	Can exercise for 30 - 40	□	□	□	□	□

Dimension	Serial Number	Item Content	Can't Do at All (1 point)	Mostly Can't Do (2 points)	Can Do Half (3 points)	Mostly Can Do (4 points)	Can Do Completely (5 points)
		minutes a day as required.					
	5	Can do warm - up exercises before each exercise as required.	□	□	□	□	□
	6	Can do relaxation exercises after each exercise as required.	□	□	□	□	□
Exercise Monitoring Compliance	7	Can monitor one's own pulse during exercise as required.	□	□	□	□	□
	8	Can record each exercise diary as required.	□	□	□	□	□

Dimension	Serial Number	Item Content	Can't Do at All (1 point)	Mostly Can't Do (2 points)	Can Do Half (3 points)	Mostly Can Do (4 points)	Can Do Completely (5 points)
	9	Can observe the improvement of fatigue by oneself.	□	□	□	□	□
	10	Can urge oneself to exercise every day.	□	□	□	□	□
Active Seeking of Advice Compliance	11	Can accept the supervision of family members or friends during exercise.	□	□	□	□	□
	12	Can accept the supervision of medical staff or professionals during exercise.	□	□	□	□	□
	13	Can communicate with	□	□	□	□	□

Dimension	Serial Number	Item Content	Can't Do at All (1 point)	Mostly Can't Do (2 points)	Can Do Half (3 points)	Mostly Can Do (4 points)	Can Do Completely (5 points)
		medical staff when problems arise during exercise.					
	14	Can communicate with fellow patients who also need to do exercise training.	□	□	□	□	□
	15	Can actively seek suggestions to improve the effect of exercise training when the effect of exercise training is not obvious.	□	□	□	□	□
